# Crystallographic analysis of the lattice metric (*CALM*) from single electron backscatter diffraction or transmission Kikuchi diffraction patterns

**DOI:** 10.1107/S1600576721004210

**Published:** 2021-05-28

**Authors:** Gert Nolze, Tomasz Tokarski, Łukasz Rychłowski, Grzegorz Cios, Aimo Winkelmann

**Affiliations:** a Federal Institute for Materials Research and Testing (BAM), Unter den Eichen 87, 12205 Berlin, Germany; b TU Bergakademie Freiberg, Institut für Mineralogie, Brennhausgasse 16, 09599 Freiberg, Germany; c AGH University of Science and Technology, Academic Centre for Materials and Nanotechnology, Mickiewicza 30, 30-059 Krakow, Poland; d Department of Physics, SUPA, University of Strathclyde, Glasgow G4 0NG, United Kingdom

**Keywords:** electron backscatter diffraction, Kikuchi patterns, lattice parameters, Radon transform

## Abstract

A new software is presented for the determination of crystal lattice parameters from the positions and widths of Kikuchi bands in a diffraction pattern. Starting with a single wide-angle Kikuchi pattern of arbitrary resolution and unknown phase, the traces of all visibly diffracting lattice planes are manually derived from four initial Kikuchi band traces via an intuitive graphical user interface. A single Kikuchi bandwidth is then used as reference to scale all reciprocal lattice point distances. Kikuchi band detection, via a filtered Funk transformation, and simultaneous display of the band intensity profile helps users to select band positions and widths. Bandwidths are calculated using the first derivative of the band profiles as excess-deficiency effects have minimal influence. From the reciprocal lattice, the metrics of possible Bravais lattice types are derived for all crystal systems. The measured lattice parameters achieve a precision of <1%, even for good quality Kikuchi diffraction patterns of 400 × 300 pixels. This band-edge detection approach has been validated on several hundred experimental diffraction patterns from phases of different symmetries and random orientations. It produces a systematic lattice parameter offset of up to ±4%, which appears to scale with the mean atomic number or the backscatter coefficient.

## Abstract 2

New software and algorithms for the accurate measurement of crystal lattice parameters from Kikuchi bands in a diffraction pattern are presented.

## 1. Introduction   

Up to now, the determination of crystal lattice or structure parameters from Kikuchi diffraction (KD) patterns has been a topic that has rarely moved into the focus of interest. The reason for this is the high expectation of users who compare results with the standard method for lattice parameter determination, X-ray diffraction (XRD). Meanwhile the high automation of XRD techniques makes one forget that even this method has only been automated in the past 2–3 decades to such an extent that users should be able to obtain highly accurate and reliable data after some practice.

Bragg angles extractable from electron backscatter diffraction (EBSD) or transmission Kikuchi diffraction (TKD) patterns are significantly smaller and less precise than those of the frequently used Cu *K*α radiation in XRD owing to the considerably shorter wavelength of commonly used electron energies. However, the application of electron diffraction is not to be seen as in competition with XRD, but rather as an option when XRD as a method is no longer able to provide the desired results. This case occurs mainly when the fraction of the phase to be determined falls below a reliably measurable value. It is only when minimal precipitates are to be characterized that electron diffraction becomes interesting, and even general statements about the symmetry of the phase or the order of magnitude of the lattice parameters are often sufficient to draw conclusions about the nature of the precipitating phase.

This task has been covered for decades by transmission electron microscopy (TEM), which with diffraction methods such as selected-area electron diffraction (SAED) produce a point pattern equivalent to the distribution of lattice points in reciprocal space (Dorset, 1995 ▸; Zou *et al.*, 2004 ▸, 2011 ▸; Lábár, 2005 ▸; Lin, 2014 ▸; Yang *et al.*, 2017 ▸; Li, 2019 ▸). Several of these point patterns as sections through the reciprocal space are necessary to estimate the Bravais lattice type, the crystal system expected from it and, roughly, the lattice parameters. TEM makes it possible to obtain diffraction information from even the smallest crystals, which in XRD would produce broadened reflections (increased peak widths caused by the small grain size, not to mention the small phase amount) due to the longer wavelength, making XRD unsuitable as an investigating method.

But TEM also has certain disadvantages, for example the need to take several diffraction patterns of the same crystallite from different angles, which quickly becomes extremely experimentally challenging. One solution is electron diffraction tomography (Kolb *et al.*, 2008 ▸; Zhang *et al.*, 2010 ▸).

Another requirement is that the samples have to be electron transparent, *i.e.* a preparation of very thin samples (about 100 nm) is necessary, which reduces the probability of finding the desired phase in sufficient quantity in the remaining specimen volume. This problem at least could be solved by target preparation techniques like the use of a focused ion beam.

Therefore, even during the early research on EBSD, there was hope that this technique could also be used for locally resolved phase analysis or identification. Over the years there have been repeated attempts to reevaluate the possibilities of lattice parameter or symmetry determination, often after introduction of improved detectors (Baba-Kishi & Dingley, 1989 ▸; Goehner & Michael, 1996 ▸; Michael & Eades, 2000 ▸; Dingley & Wright, 2009 ▸; Saowadee *et al.*, 2017 ▸), but these were never followed by commercial success, so that a further dissemination of the developed approaches failed to materialize. In contrast to this, the belief has prevailed that EBSD is a technique which can be used very well for the fast analysis of orientation and phase distribution but which seems completely unsuitable for a crystallographic phase characterization. This is astonishing, because the diffraction patterns of different structures can look very diverse. One reason for the lack of faith may be that a physically based simulation of diffraction patterns was published very late (Winkelmann *et al.*, 2007 ▸). It took another 12 years until the intensity information of such simulations was used for the first time to improve the indexing of KD patterns (Wright *et al.*, 2019 ▸). So far, all EBSD systems have worked with purely geometrically derived band positions and have been based on the kinematic diffraction theory, which is actually unsuitable for electrons because individual reflectors are used for indexing instead of the entire band information.

The most promising approach for lattice parameter determination and Bravais lattice type derivation so far was provided by a Chinese group around Ming Han. They showed with the software *EBSDL* (Li *et al.*, 2014 ▸) that with only information on the electron wavelength and a single KD pattern a derivation of the crystal lattice is possible (Li & Han, 2015 ▸; Han *et al.*, 2018 ▸; Han & Zhao, 2018*a* ▸,*b* ▸). This includes not only the lattice parameters and Bravais lattice types derived from the band positions and widths but also the simultaneous determination of the projection centre. If the projection centre position is correct, the fit between the band positions and widths given on the diffraction image and the crystal and measurement quantities derived from them are perfect.

We take up this idea and show that the derivation of the lattice metric from a single KD pattern can be greatly simplified by the specific application of some basic crystallographic principles. This reduces possible errors and is furthermore associated with significantly lower requirements on the resolution of the diffraction patterns as well as the monitor used.

In addition, new visualization tools make the systematic relationships between diffraction patterns and crystallographic laws and rules easier to understand than the approach used in *EBSDL* of minimizing deviations between individually and independently described band positions and widths and a possible crystal lattice.

We assume, in contrast to *EBSDL*, that the projection centre is sufficiently known to a first approximation, for example determined from a pattern corresponding to a known phase of the surrounding matrix.

## 2. General functionality   

### 2.1. Diffraction pattern projection   

**Figure 1:**
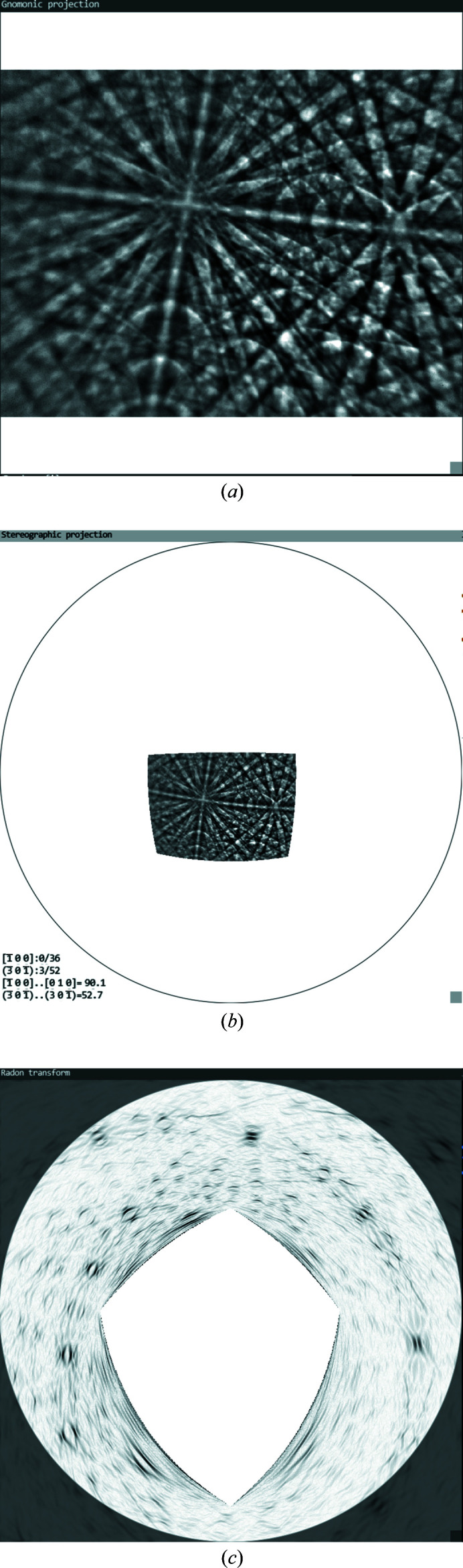
Different projections in *CALM* of a diffraction pattern of cassiterite (SnO_2_): (*a*) gnomonic projection, (*b*) stereographic projection and (*c*) edge-filtered Funk transform.

*CALM* is a C++ program with its own graphical user interface. It displays the KD pattern as it was originally recorded by the detector and as background-corrected by the used acquisition software. Although the image can be captured by either a round or a rectangular detector screen, the latter has the advantage of using the entire image area of a CCD or CMOS chip [see Fig. 1 ▸(*a*)]. Therefore, the captured sector is effectively larger.

Besides the gnomonic projection as the central projection from a point source onto a flat plane, the KD pattern is also displayed in stereographic projection using the projection centre (PC) position as origin [see Fig. 1 ▸(*b*)]. Among other advantages, this presentation makes the captured sector size visible.

**Figure 2:**
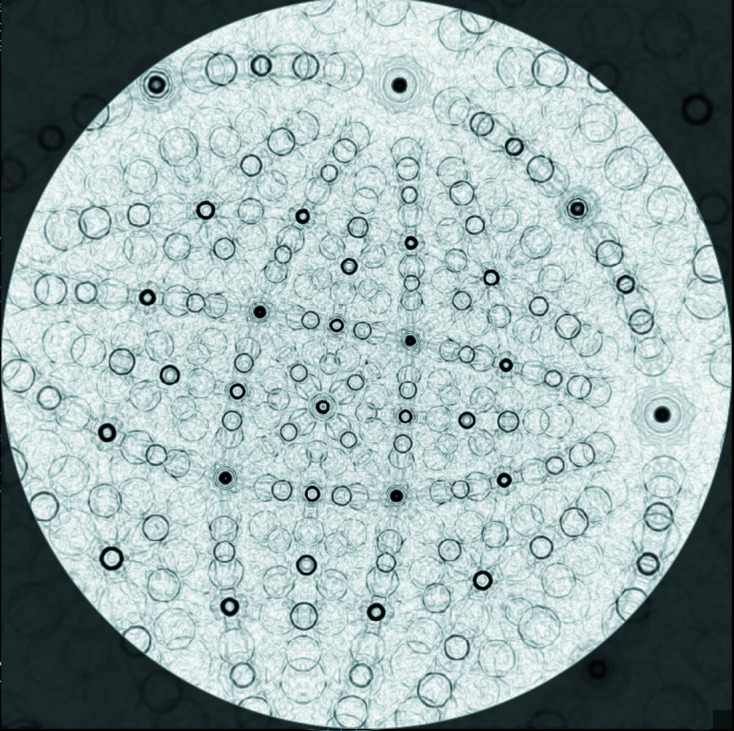
Stereographic projection of the edge-filtered Funk transform of the full Kikuchi diffraction signal simulated for the same orientation of cassiterite (SnO_2_) [*cf*. Fig. 1 ▸(*c*)].

A fully automated detection of bands as equivalents for traces of diffracting lattice planes is only possible to a limited extent from KD patterns. The search for an acceptable tool ended with the introduction of the Hough transform, which modifies the line-like into point-like intensity features whose maxima are comparatively easy to determine (Krieger Lassen, 1992 ▸). Unfortunately, typical crystallographic properties such as the affiliation of lattice planes to a zone are no longer so obvious. Therefore, we decided to use an alternative transformation, which to our knowledge was first applied to EBSD patterns by Day (2008 ▸). It is based on the Funk transform (Funk, 1915 ▸), often also called the spherical Radon transform, which integrates the diffracted intensity projected on a sphere along great circles. Displayed as a stereographic projection and modified by appropriate edge filtering and intensity adjustment, it makes the bands appear as rings (*cf*. Fig. 2 ▸).

In the case of an incomplete diffraction signal such as a KD pattern, the bands form degenerated rings, which are, however, still lined up along a great circle when they belong to a zone. The degeneration becomes stronger the smaller the solid angle detected by the camera. When the bands in the diffraction pattern become even shorter at corners or outer edges, the typical (half) rings look more like an eye that increasingly degenerates into two parallel lines (Fig. 1 ▸). But their distance is still equal to the diameter of the ideal circle which indicates the bandwidth.

A major advantage of the adapted Funk transform is that up to twice as many bands can be detected, especially wide bands that are difficult to identify in gnomonic projection but very beneficial for the determination of lattice parameters.

### 2.2. Fixing the lattice plane position   

It is generally accepted that the centres of Kikuchi bands mark the imaginary intersection of infinitely extended diffracting lattice planes (*hkl*) with the detector screen. This is not entirely true, because the gnomonic distortion leads to the fact that with increasing distance from the so-called pattern centre (orthogonal projection of PC onto the detector screen) the displacement of the intersection line from the local band centre increases. This means that the assumption that the Hough peak represents the normal direction of the diffracting lattice plane is suitable for a rough orientation determination but too vague for more advanced analytical techniques like the derivation of lattice parameters.

**Figure 3:**
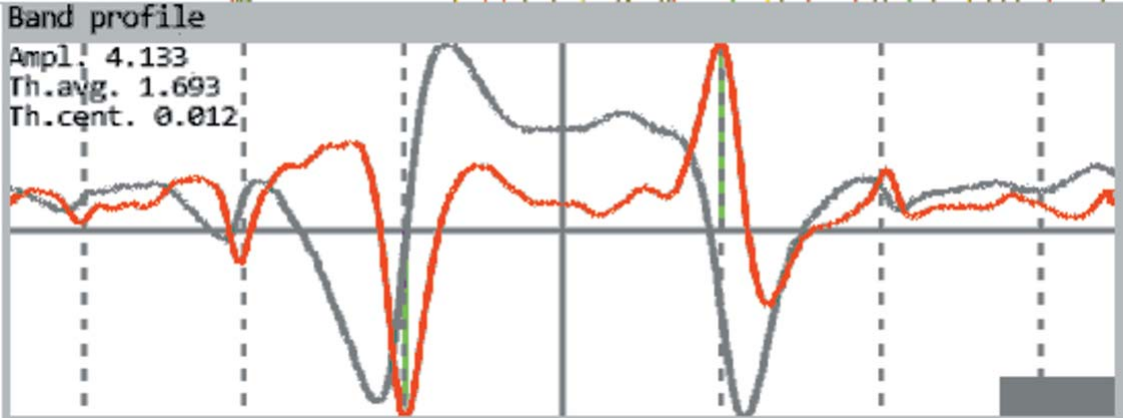
Band profile (grey) and derived band edge positions (green vertical lines) given by the extrema of the first derivative (red curve) (Saowadee *et al.*, 2017 ▸). The vertical dashed lines are the adapted Bragg angles for the first reflection orders. Ampl. describes the maximum amplitude of the profile. The Bragg angle (Th.ang.) and the asymmetry of the extremum positions (Th.cent.) displayed by the green lines are given in degrees.

On the other hand, the point of intersection of lattice plane traces marks a lattice direction [*uvw*], which is the zone axis. Lattice planes belong to the same zone when they have an identical zone axis, *i.e.* their traces meet at a point. This concept describes the trace definition using the four-line approach formulated by Nolze & Winkelmann (2017 ▸) for EBSD. It states that by fixing only four traces, no three of which are tautozonal, the traces of all other visible bands can be inferred without doubt. Thus the error in the trace position is determined by only these four initial traces. Conversely, the setting of the four initial traces can be significantly enhanced by improved matching of all other traces in this way, and most importantly, gnomonic distortions are easy to recognize and to correct. This approach is further extended by another tool which represents the intensity summation 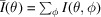 and gives the intensity profile of the band as function of the diffraction angle θ (*cf*. the grey curve in Fig. 3 ▸).

The band edge positions (extrema of the red curve indicated by the green lines) are only symmetric to the trace if the trace is centred with respect to the diffraction angle θ. The shift out of the centre is given in Fig. 3 ▸ by the value Th.cent., which is for the displayed band +0.012°. Both conditions together, the qualitative match of all band positions and the quantitative deviation Th.cent., allow a sufficient description of the lattice plane traces by shifting and tilting the initial traces only, even for low-resolution patterns.

The match between experimentally derived and ideal lattice planes in 3D depends not only on the trace description but also on a correct position of the projection centre PC. Since PC is always subject to small errors, lattice planes can never be exactly described, even if the trace positions appear to be perfect.

The manual setting of the four initial lattice planes is possible in the gnomonic projection, where four straight lines must be drawn as their traces, as well as in the filtered Funk transformation, where the centres of circles can be roughly described as the direction of the normal vector by a double-click on the mouse button.

**Figure 4:**
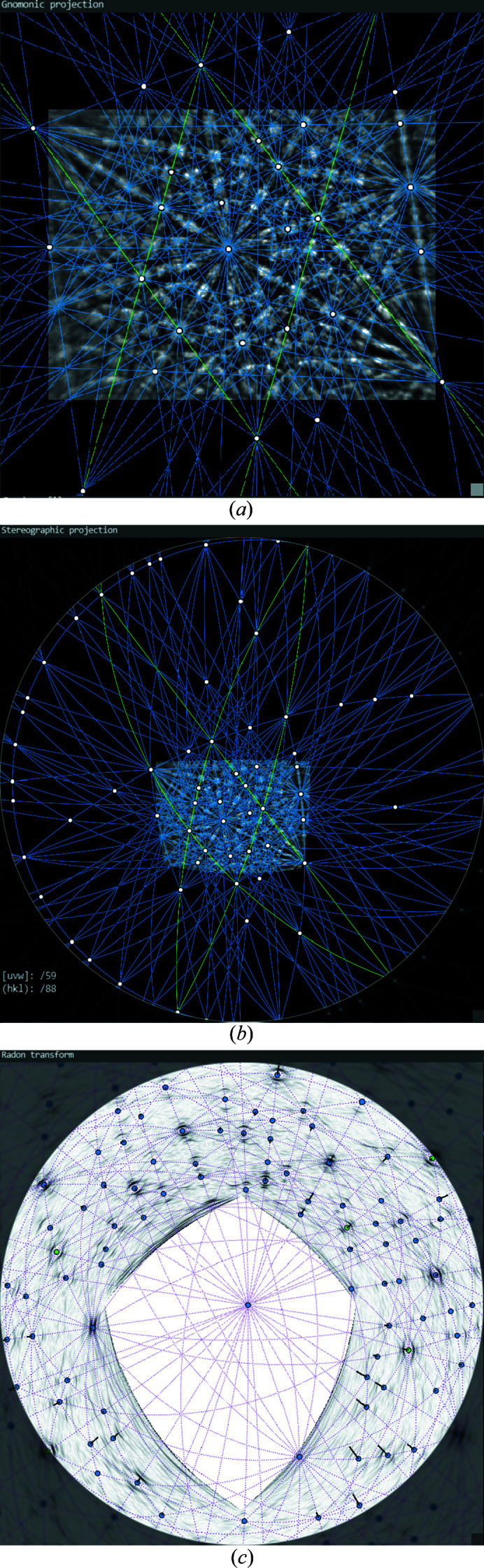
Different options to represent crystal lattice features: (*a*) gnomonic projection, (*b*) stereographic projection and (*c*) edge-filtered Funk transform. In (*a*) and (*b*) all zone axes are marked by white dots where more than two lattice planes (blue lines) intersect each other. Green lines indicate the four initial traces. In (*c*) the blue dots indicate the normals to the lattice planes and the dotted lines represent zone axes.

The derivation of other lattice planes from the four initial traces, however, is possible in all three projections: gnomonic and stereographic projections of the pattern as well as in the Funk transformation. This is done by combining declared lattice directions [*uvw*], whereby newly usable [*uvw*] are created by the intersections of already fixed lattice planes. In the stereographic projection in Fig. 4 ▸(*b*), main zones are characterized by low-indexed zone axes aligned along great circles. These zone axes describe again zones of several bands. From Fig. 4 ▸(*b*) it is also clear that a KD pattern contains practically all information about the entire crystal lattice, since the majority of the used zone axes and lattice planes lie far outside the region represented by the KD pattern. Thus, possible symmetries can be analysed even if they are not visible directly in the KD pattern.

In the edge-filtered Funk transform in Fig. 4 ▸(*c*) planes and directions appear opposite. Lattice planes are represented by directions (blue dots), while zone axes become great circles (thin dotted lines) along which all band-forming lattice planes of this zone are aligned. Asymmetric positions of the extrema of the first derivative are displayed as black lines for each given lattice plane. The direction of the black line points to the projection centre, whereas the sign of the angular shift determines whether the line points to or away from the centre. Systematic deviations, as visible in the lower right sector where all lines are directed toward the centre, indicate a slightly imperfect positioning of at least one of the four initial traces. A slight readjustment can help to reduce such systematic shifts.

All three available possibilities of fixing the lattice planes have their advantages and disadvantages. The gnomonic projection allows a rather simple description of lattice plane traces but is unbeatable especially during visual alignment and readjustment of the four initial traces. The human brain is optimized to recognize deviations from parallel alignments so that the gnomonic projection is perfect for fine-tuning. In the case of many overlapping bands, however, most errors occur there, because despite the adjustable magnification of the angular range [zoom-out compared with Fig. 1 ▸(*a*)] similarly aligned bands can easily be mistaken for each other.

The stereographic projections in Figs. 4 ▸(*b*) and 4 ▸(*c*), on the other hand, offer more systematic approaches, because there zones whose zone axes lie far outside the segment covered by the EBSD detector screen can still be reliably recognized and used. The KD pattern shown in Fig. 4 ▸(*b*), however, only helps to a limited extent owing to its small size, so that a combined use with the gnomonic projection is recommended.

The Funk transformation in Fig. 4 ▸(*c*) is unusual because of its inverted character: traces become points and zone axes (points) become great circles. Apart from the lower suitability for the alignment of the four initial traces, it is, however, the most universal possibility for an unequivocal detection of lattice planes. In particular, it shows the bands of lattice planes that one would never find in a gnomonic projection even if there was no strong overlapping of bands. In experimental KD patterns of complex but nearly perfect crystal structures (*e.g.* metal alloys) we discovered up to 160 bands. For the pattern in Fig. 4 ▸ more than 130 bands can be identified.

**Figure 5:**
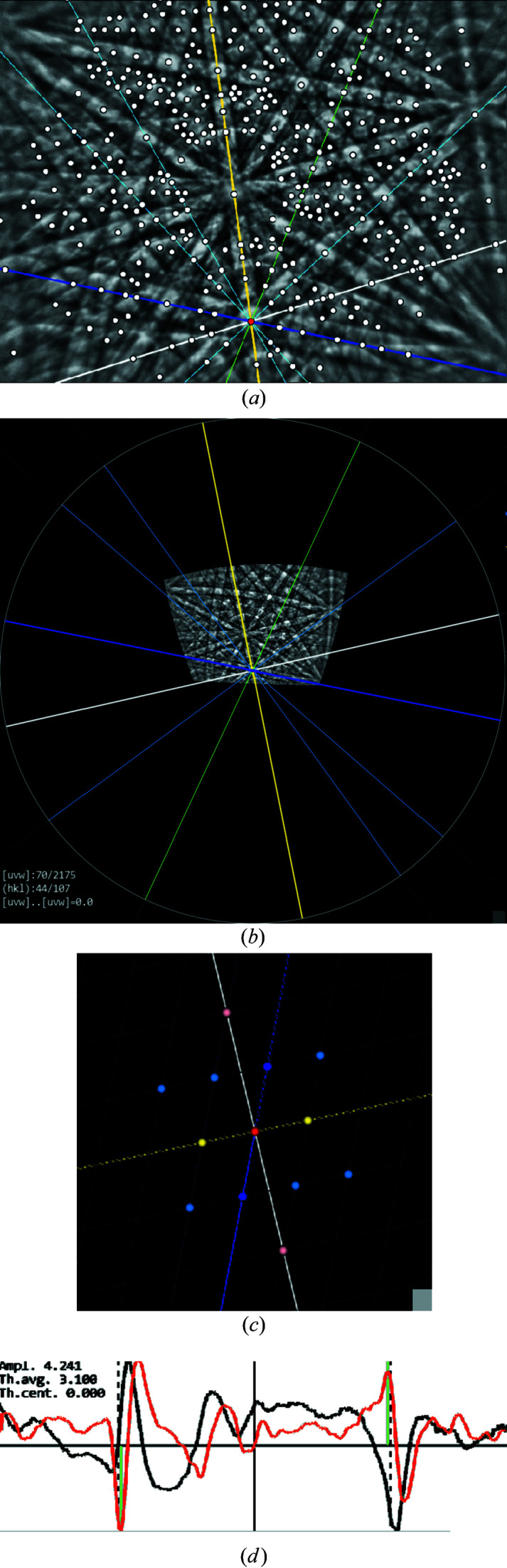
Bandwidth selection using the gnomonic projection (*a*). Here, all zone axes are marked by white dots where more than two described lattice planes intersect. After rotation to the centre of the stereographic projection (*b*), only a limited number of solutions result which satisfy the translation symmetry of the reciprocal lattice plane (*c*). Whether the trace actually represents a lattice plane of the zone can be recognized by its alignment. Which interference order is then relevant has to be decided on the basis of the band profile (*d*).

Misinterpretations can be almost completely eliminated by displaying the band profiles simultaneously (*cf*. Fig. 3 ▸). If Th.cent. is greater than for adjacent bands, the probability is very high that an incorrect lattice plane has been assigned to a zone. Similar situations can occur if a pattern is unusually noisy. Therefore, the derived band profile is also displayed so that the user can visually decide whether the deviation is of a systematic nature or results from a band profile shape that is influenced by other strong bands [*cf*. the profile drawn in black in Fig. 5 ▸(*d*)] which affect the band profile by superimposition.

### 2.3. Bandwidth selection   

The relative metric of the crystal lattice is in practice already fixed by the trace positions and the assumed projection centre PC (Nolze & Winkelmann, 2017 ▸). This means that the ratios of the lattice parameters as well as the angles between the basis vectors are already now invariant. All directions of the crystal lattice and of the reciprocal lattice are fixed, although their specific indexing is not yet known since the basis vectors still have to be found. For fixing the absolute lengths of the basis vectors we only need a single reference bandwidth, which is equivalent to the distance of a reciprocal lattice point from the origin (Nolze & Winkelmann, 2017 ▸). The necessary conditions for all other reciprocal lattice point positions are given by the combination of lattice directions and the translation symmetry, which is of course also valid for the reciprocal lattice. All previously fixed directions and planes must, without exception, be describable by newly discovered lattice and reciprocal lattice points. From the derived reciprocal lattice vectors the reduced cell can be deduced, which enables the determination of the primitive basis vectors of the crystal lattice, *e.g.*

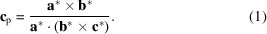
From the basis vectors 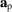, 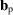, 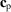 and the angles α_p_, β_p_, γ_p_ between them the most probable Bravais lattice types and their lattice parameters *a*, *b*, *c*, α, β, γ are derivable [see *e.g.* Gruber (1973 ▸, 1989 ▸)].

In *CALM* the reference bandwidth can be set at any band and can be adjusted at any time, for example to a band that seems more suitable than the band originally selected.

The determination of the band edge by the first derivative was proposed by Saowadee *et al.* (2017 ▸) for an automated tracking of bandwidths. In Figs. 3 ▸ and 5 ▸(*d*) the first derivative is displayed as a red curve. In *CALM* it mainly serves as a decision criterion for the description of a reciprocal lattice point. If correct, the extrema of the first derivative show a perfect match with the expected Bragg angle positions θ_B_ derived from the width of the reference band and drawn as vertical dashed lines.

In Fig. 5 ▸ the crystallographic derivation of a bandwidth is demonstrated for a selected zone defined by intersecting lattice plane traces. The red point marks the projection of the zone axis [*uvw*]. To select the width of the band with the white trace, the bands with the yellow and blue coloured traces are used. The developed procedure searches for the combination of reciprocal lattice vectors which, on the one hand, satisfies (or adapts) the translation symmetry of the lattice already known in this zone and, on the other hand, correctly represents the interference order(s) visible through the band profile (Nolze & Winkelmann, 2017 ▸). There are only a very limited number of combinations that fulfil both conditions. Zone axes with only three intersecting lattice planes are very successful, since it is highly likely that in the case of one known bandwidth the other bandwidths can be immediately derived by vector addition or subtraction. For a better visualization the selected zone axis can be virtually rotated into the pattern centre as shown in Fig. 5 ▸(*b*). The corresponding reciprocal lattice plane 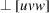 is now located in the image plane [Fig. 5 ▸(*c*)]. It contains all reciprocal lattice points of which we only know the normal directions to (*hkl*). However, we do not know the distances of the points from the origin, but we know that they must all satisfy the conditions of a translation lattice. The regular line grid, just visible in the background of Fig. 5 ▸(*c*), is derived from the shortest known reciprocal lattice vectors that have been discovered up to this point in time in this specific reciprocal lattice plane. The intersection points of the grid indicate possible positions of reciprocal lattice points. They already describe the translation symmetry either in the reciprocal lattice plane 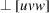 or of a sublattice. Newly derived vectors must therefore either match these lattice points or create new lattice point layers, which have to be compatible with the already existing lattice points. In Fig. 5 ▸(*c*) a yellow vector (given by the position of the left yellow sphere) and a blue direction have already been selected. The resulting intersection of the blue with the white line after a virtual shift of the blue line into the yellow sphere is marked as a pink sphere. The distance from the pink sphere to the origin represents the resulting Bragg angle θ_B_ and is given as the dashed lines in Fig. 5 ▸(*d*). In the case of a match with the extreme positions (green lines) of the first derivative the probability is very high that the bandwidth is correctly chosen. Moreover, higher interference order(s) are easy to recognize (*cf*. Fig. 3 ▸). Starting from a band that contains several zone axes and thus ensures access to all other bands, all bandwidths and thus the reciprocal lattice can be derived step by step.

### 2.4. Bravais lattice type and lattice parameter determination   

After transformation of the reciprocal lattice into a real-space translation lattice its classification is still to be determined, *i.e.* possibly matching Bravais lattice types need to be tested. This procedure uses purely metric conditions, which are described in detail elsewhere (Le Page, 1982 ▸; Macíček & Yordanov, 1992 ▸; Grimmer, 2015 ▸). Because of small errors in fixing the band traces and PC position, often a whole series of basis vector descriptions results. The number of presented Bravais lattice descriptions depends on the maximal deviations entered for α, β and γ as well as for the respective lattice parameter ratios. Although the highest-symmetry description is often the most probable (Müller, 2013 ▸), simultaneously also all lower-symmetry solutions are listed in real time. For the KD pattern of cassiterite (Fig. 5 ▸) all solutions are given in Table 1 ▸.

Owing to experimental errors (fixing the initial traces, PC) no angle between the base vectors results exactly in 90°, and the ratio between *a* and *b* is not 1 as expected but shows only a tiny deviation of −0.1%. However, even under ideal conditions, Bravais lattice solutions can differ considerably from the common lattice descriptions. For orthorhombic crystals, for example, the basis vectors can be permuted without restriction, which naturally leads to different sets of lattice parameter ratios *a*/*b*:1:*c*/*b*. This seems surprising when used to identify minerals in databases.

**Figure 6:**
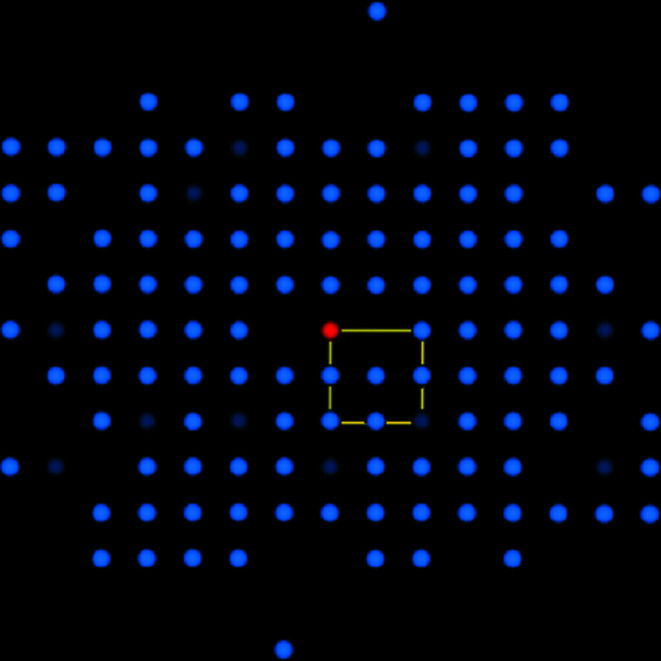
Derived reciprocal lattice points projected along [001]. The unit cell in reciprocal space (yellow frame) is all face centred which indicates a body-centred unit cell in real space. Dark-blue spheres represent higher-order interferences. The red sphere denotes the origin 000.

Therefore, *CALM* contains a tool that is valuable for the identification of potentially erroneous Bravais lattice solutions caused by wrong band descriptions and therefore incorrect reciprocal lattice points. It displays all derived reciprocal lattice points in 3D. Fig. 6 ▸ shows the reciprocal lattice of an all-face-centred unit cell F*, which is outlined by the yellow frame. F* always corresponds to a body-centred point lattice in real space: F* ↔ I. If single bandwidths are incorrectly described, the derived reciprocal lattice points do not fit the translation symmetry of all other lattice points. Incorrectly assigned bandwidths that do not fit the translation symmetry of the reciprocal lattice inevitably produce a larger unit cell.

Fig. 6 ▸, however, only displays correct reciprocal lattice points. Missing points are irrelevant. They simply indicate non-detected bands. The omnipresent translation symmetry is assumed to fill all the gaps where points are missing.

### 2.5. Reducible and unavoidable errors   

#### 2.5.1. Experimental errors   

The different Bravais lattice solutions in Table 1 ▸ indicate subgroup relationships between the lattices (Müller, 2013 ▸). The errors given in brackets are only estimates. They are based on a possible misalignment of the lattice plane traces and simply describe the variation of the lattice parameters when the bandwidth and band alignment are changed. Since the four initial traces are set manually, future improvements can only be expected by automatically adjusting the traces to minimize the errors.

A modification of the projection centre position of, for example, ΔPCx = 0.001 would cause a change in the lattice parameters of up to |Δ*a*| = 0.004 Å, which results in a variation of the lattice parameter ratios of |Δ(*a*/*b*)| = 0.001. The derived lattice parameters or lattice parameter ratios change by a comparable amount in response to a shift or tilt of the lattice plane traces. Owing to the large number of bands taken into account, the precision of the lattice parameter ratios is virtually unchanged even with less well resolved Kikuchi patterns.

#### 2.5.2. Accuracy and precision   

**Figure 7:**
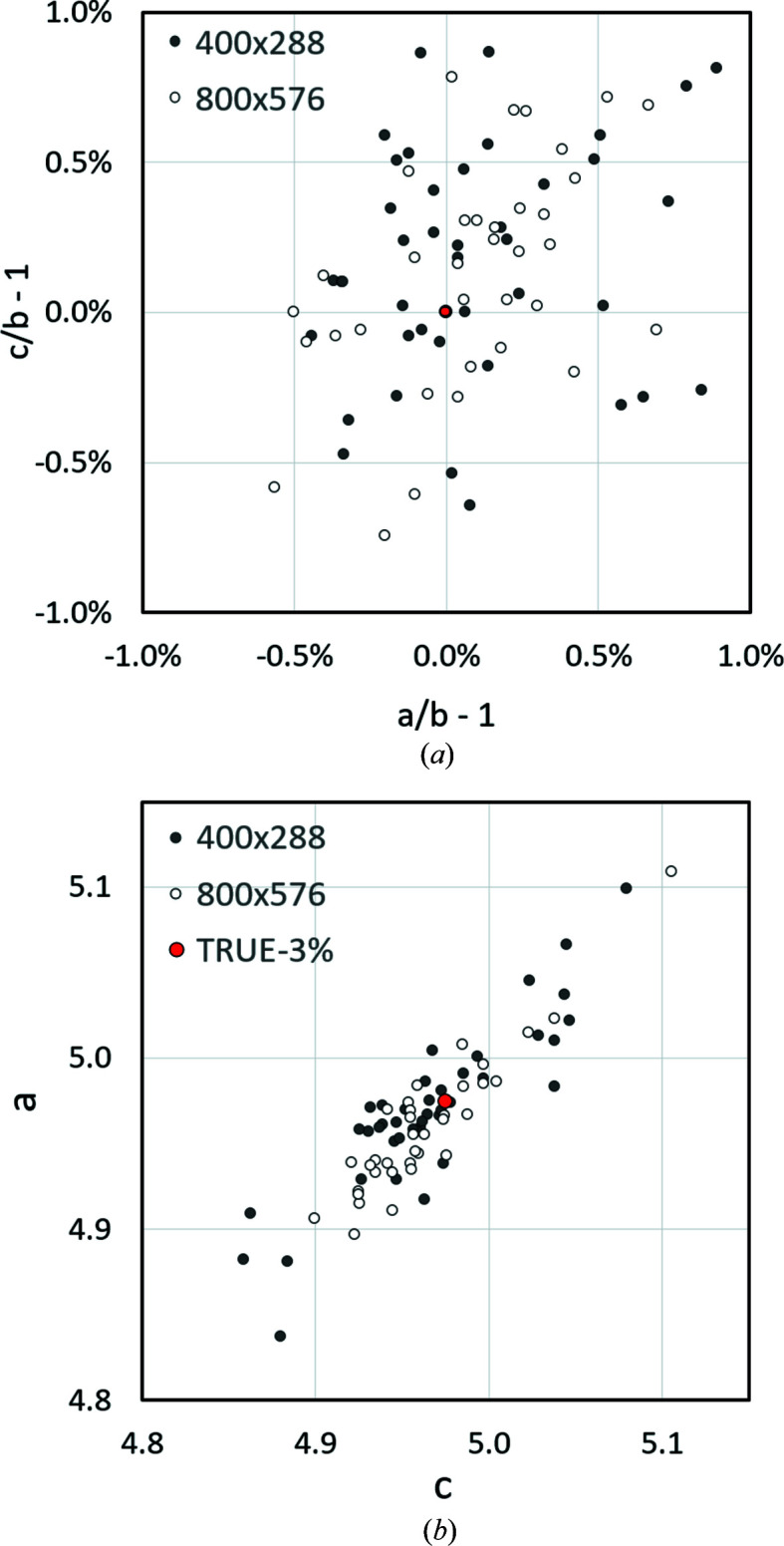
Analysis of 41 patterns of rhombohedral corundum (Al_2_O_3_) collected with a resolution of 400 × 288 pixels and 34 patterns of 800 × 576 pixels. (*a*) Displays the deviation of *a*/*b* and *c*/*b* from 1, whereas (*b*) presents the absolute lattice parameters derived for *a* and *c* (*b* as well as α, β and γ are not shown). The red dot shown in (*b*) and labelled TRUE-3% marks a 3% shorter basis vector length than that typical for corundum (*a* = 5.1288 Å).

To better estimate the accuracy and precision, 34 higher-resolution (800 × 576 pixel) and 41 lower-resolution (400 × 288 pixel) KD patterns of corundum (Al_2_O_3_, rhombohedral) were examined, all showing arbitrary orientations. The results in terms of both the lattice parameter ratios (*a*) and the absolute lattice parameters (*b*) are shown in Fig. 7 ▸. With purely manual trace positioning, higher-resolution KD patterns do not automatically lead to a significant improvement in the precision of the lattice parameters [Fig. 7 ▸(*a*)]. The reason is the already discussed bandwidth, which can be evaluated for corundum almost loss free even with lower-resolution patterns. Furthermore, it should not be forgotten that only a single bandwidth is necessary, while the others are only used for the discrimination of the Bravais lattice type. This means that also for phases with narrower bands only a single wide band affects the absolute scaling of the lattice parameter. Since the widths of the reference bands in all patterns were comparable, the uncertainty in the derived lattice parameters is practically identical. The accuracy of the lattice parameter ratios is in all probability even highe, since position and slope are in principle better fixable for narrow bands than for wide bands.

While the precisions of the lattice parameter determinations on corundum performed on 75 samples are comparable to one another and better than expected for both pattern resolutions, *cf*. the deviation from the diagonal in Fig. 7 ▸(*b*) of approximately ±0.025 Å (∼ ±0.5%), the accuracy of approximately ±0.1 Å (∼ ±2%) is somewhat lower. But it is also much better than expected, especially if we consider the low pixel resolution of some of the patterns.

More worrying is the size of the absolute lattice parameter determined, which is consistently ∼3% short. This observation suggests that the bandwidth determination by the first derivative does not correlate exactly with the Bragg angle. Further reasons for the observed offset could be excess-deficiency effects, which might influence the accuracy of the projection centre determination systematically. Therefore, an alternative determination of the projection centre based on four indexed lattice directions [*uvw*] was applied (Nolze *et al.*, 2020 ▸). This actually reduced the deviation from the ideal lattice metric but did not eliminate the observed shift.

#### 2.5.3. Lattice parameter offset   

In order to prevent any uncertainty resulting from varying lattice parameter ratios, for the investigation of the nature of the observed offset only experimental KD patterns of cubic phases and random orientation were analysed. Table 2 ▸ lists the 23 cubic phases for which the lattice parameters were determined from KD patterns, some of them with considerably different image resolutions.

The phases are ordered according to the backscatter coefficient η, which is calculated by a polynomial using the atomic number *Z* (Reuter, 1972 ▸; Reimer, 1998 ▸): 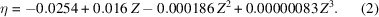
For compounds, the mean value *Z*_m_ has been taken, which is corrected by the atomic mass of the respective elements and the stoichiometry. As an alternative correlation, the mean atomic number *Z*_at_ derived from stoichiometry only is also listed in Table 2 ▸ but not shown as a separate graphic.

**Figure 8:**
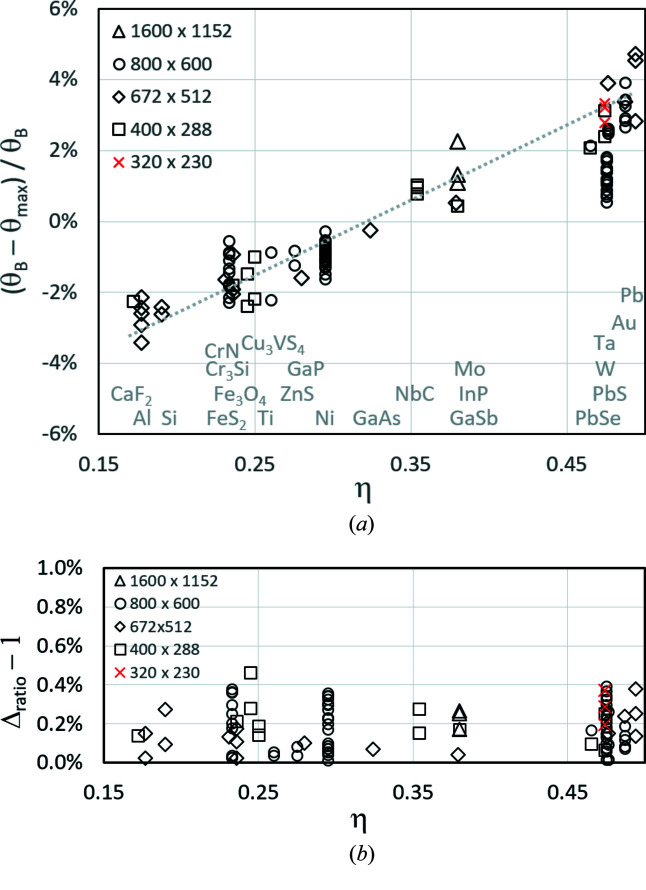
(*a*) The deviation of the experimentally derived reference Bragg angle θ_max_ (first derivative) from the Bragg angle θ_B_ computed using the lattice parameters given in Table 2 ▸. (*b*) The averaged lattice parameter ratio Δ_ratio_ = (*a*/*b* + *c*/*b*) demonstrates the low deviation from the cubic metric. For the analysis, different pattern resolutions have been used (*cf*. legend and Table 2 ▸).

In Fig. 8 ▸, the relative deviations between Bragg angles determined experimentally from the first derivative and computed theoretically from databases are shown as a function of the backscatter coefficient η.

Fig. 8 ▸(*a*) indicates that the deviation between the extremum positions of the first derivative θ_max_ and the Bragg angle θ_B_ computed from lattice parameters in Table 1 ▸ is different from phase to phase but apparently scales with η. Only for phases with η ≃ 0.35 like GaAs (η = 0.324), BaSO_4_ (η = 0.356) or SnO_2_ (cassiterite, η = 0.372) does the first derivative approximately deliver the absolute lattice parameters correctly. For phases with lower backscatter coefficients the first derivative slightly overestimate θ_B_, *i.e.* *a* < *a*_o_, while for stronger scattering phases θ_B_ is slightly underestimated and the derived lattice parameters *a*, *b* and *c* thus appear proportionally larger. Fig. 8 ▸(*b*) proves that the averaged lattice parameter ratio is not affected, which is no surprise since the ratios do not depend on the bandwidths. Moreover, compared with Fig. 7 ▸(*a*) the deviations tend to be smaller because of the higher redundancy for phases of cubic symmetry. Slightly misaligned traces are typically easier to recognize.

Fig. 8 ▸ again shows that a high KD pattern resolution does not necessarily lead to an improved accuracy of the lattice parameter determination. It is definitely an advantage, if only because of the resulting lower noise in the band profiles, but smaller image formats obviously provide sufficiently accurate lattice parameters. However, if the image quality is improved by a suitable averaging of low-resolution KD patterns, the results are practically equivalent to those derived from high-resolution KD patterns.

#### 2.5.4. Pseudosymmetry   

Table 1 ▸ suggests that the derived lattice point arrangement can be described by different Bravais lattice types. The geometrically necessary conditions are discussed in detail by Grimmer (2015 ▸) and Flack (2015 ▸) and explain why in Table 1 ▸ not only *tI* but also the subgroups *oF*, *oI*, *mS* and *aP* are listed. It cannot be excluded that, for example, an apparently hexagonal pattern is not actually formed by an orthorhombic phase. In order to control the consideration of different lattice descriptions, the maximum accepted deviations for angles between basis vectors and for symmetry-equivalent basis vector lengths can be varied in *CALM*.

Comparing the XRD reference data with the derived tetragonal cell dimensions in Table 1 ▸, *CALM* obtained lattice parameters which are about 1% larger than expected: Δ*a*/*a* ≃ 1.1% and Δ*c*/*c* ≃ 0.9%. Since for SnO_2_ η = 0.366, this deviation is also expected from the linear approach in Fig. 8 ▸(*a*).

In contrast to the absolute lattice parameters, their ratio shows a much better match:Δ(*c*/*a*)/(*c*/*a*) ≃ −0.16%. Therefore, for non-cubic phases, lattice parameter ratios are often much better suited for phase identification with EBSD than the derived lattice parameters themselves (Dingley & Wright, 2009 ▸). It is therefore only important to know the projection centre position and to have very well described lattice plane traces. Bandwidths are of minor worth.

**Figure 9:**
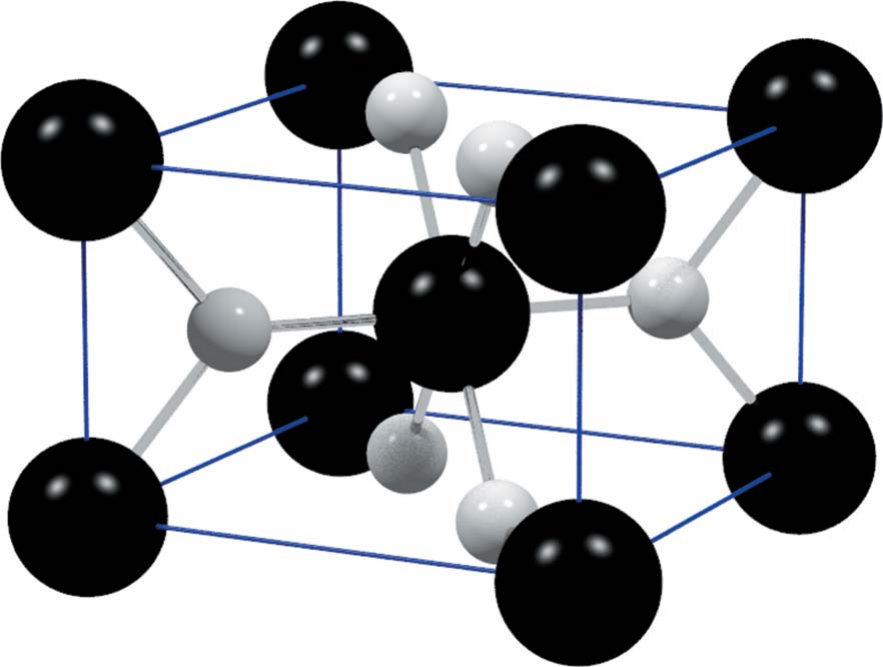
The crystal structure of cassiterite (SnO_2_) reflects the symmetry of space-group type *P*4_2_/*mnm*. The Kikuchi signal is, however, dominated by the Sn atoms, which suggests a description by a body-centred tetragonal (*tI*) lattice.

Although the lattice parameters for cassiterite are sufficiently extracted by *CALM*, a careful look at Table 1 ▸ shows that the Bravais lattice type is not correctly identified. Instead of the primitive *tP*, a body-centred tetragonal *tI* lattice has been identified (*cf*. Fig. 6 ▸). This misinterpretation has its origin in the crystal structure of cassiterite (see Fig. 9 ▸).

**Figure 10:**
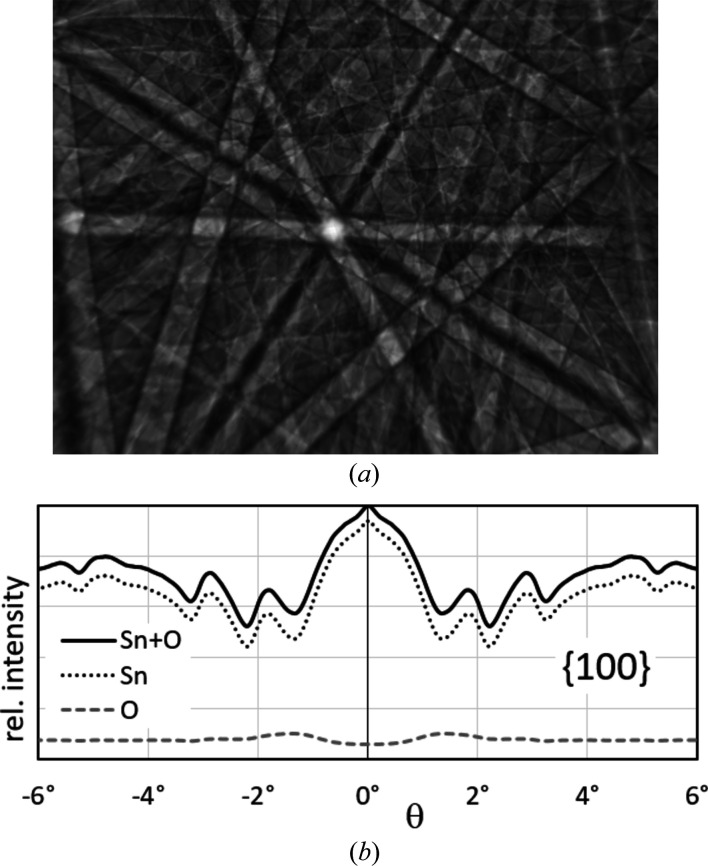
Simulated contribution (*a*) of oxygen to the total signal of SnO_2_ [*cf*. Fig. 5 ▸(*a*)]. According to the 110 profile in (*b*) the O signal is about ten times weaker than the Sn signal so that the distribution of Sn dominates the total signal.

Sn (*Z* = 50) is much heavier and occupies the same position as the lattice points in *tI*. Only the remaining O atoms are responsible for the clearly lower symmetry. O atoms (*Z* = 8) have, however, a significantly lower scattering power so that the visible diffraction signal used in *CALM* is actually dominated by Sn but considers of course the whole structure of cassiterite. The dynamical simulation of Kikuchi patterns reported by Winkelmann *et al.* (2007 ▸) allows one to separate the intensity contributions of each individual element to the overall pattern. The extracted intensity profiles of Sn and O in Fig. 10 ▸(*b*) confirm the suspicion of a weak signal of oxygen compared with Sn. As expected, the simulated pattern in Fig. 10 ▸(*a*) reveals the correct Bravais lattice type *tP*.

The example of SnO_2_ shows that a diffraction signal can fake higher translation symmetry if low-intensity bands are difficult to identify and therefore reciprocal lattice points are systematically hidden. In this specific case the lattice parameters are only accidentally not influenced, because all undiscovered translation vectors describe without exception centred reciprocal lattice points and therefore the basis vectors for *tI* and *tP* are identical. However, the resulting primitive, reduced cells are different. In the cassiterite pattern for approximately 1/4 of bands the interference order was wrongly assigned. Their first order actually has only half the Bragg angle as actually fixed.

Similar effects are known for ordered structures where independent atomic positions are occupied by atoms with comparable atomic number *Z*. Typical examples are pseudo-cubic chalcopyrites and stannites or intermetallics like TiAl or Ni-base superalloys. Often the ordering causes multiplied basis vectors, which results in larger unit cells. This results in bandwidths that are narrower than the base vector and longer by the same factor. However, the resulting new first order is often so weak in intensity that it is hardly detectable in the centre of the high-intensity band of the now higher interference order.

#### 2.5.5. Sample charging   

Additionally to all uncertainties related to band positioning, Bragg angle approximation and projection centre position, one effect is not considered at all: the electron energy which delivers the wavelength. *CALM* uses the acceleration voltage and the relativistic de Broglie relationship for the computation of the electron wavelength (Nolze & Winkelmann, 2017 ▸). Local charging, however, can easily reduce the energy so that the wavelength λ_e_ might be larger than assumed. If the bandwidth 2θ is taken as a measured constant, then according to Bragg’s equation for EBSD, 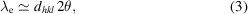
the derived *d*_*hkl*_ increases by the same factor as λ_e_ changes by the decrease of the electron energy and suggests larger lattice parameters. This means that the real lattice parameters could be proportionally shorter than those calculated by *CALM*.

## 3. Summary and conclusions   

*CALM* is a Windows software that allows the recognition of the Bravais lattice type as well as the determination of lattice parameters from a single wide-angle Kikuchi diffraction pattern, such as those obtained during EBSD or TKD measurements. The requirements for such crystal lattice analyses are

(i) an EBSD pattern of highest possible quality, but not necessarily of high resolution,

(ii) knowledge about the projection centre (PC) position (alternatively, a reference pattern of a cubic phase collected under identical conditions is suitable to derive PC in *CALM* directly),

(iii) the applied acceleration voltage, which is also used as electron energy, and

(iv) a monitor resolution of at least 1920 × 1080 pixels.

CALM has a graphical user interface and mainly uses combined keyboard and mouse interactions to fix and derive lattice planes and directions. In this way, all further planes are derived from four selected non-tautozonal planes by connecting two already existing zone axes. The derivation of the lattice planes and directions is possible in the original KD pattern, but also in the stereographic projections of the pattern or its Funk transformation. For the manual adjustment of lattice plane and direction alignment the original pattern is best, whereas for a fast and reliable fixing of trace positions the stereographic projections are recommended.

Regardless of the crystal symmetry, only one single bandwidth is required for the calculation of the lattice parameters. All other bandwidths must be describable from this bandwidth, which ultimately determines the Bravais lattice type. This and the lattice parameters are displayed during the selection of the bandwidths.

The software has been tested on hundreds of experimental and some simulated patterns for different image resolutions and for phases of different symmetries. The experience gained leads to the following initial conclusions:

(1) The developed algorithms work reliably for any crystal symmetry. Although complicated crystal structures make it difficult to fix the band positions, the selection of the bands is still straightforward.

(2) *CALM* also enables less experienced users to develop a feeling for the relationship between crystal and reciprocal lattice. Fundamental laws and rules of crystallography can be experienced visually through consistent use.

(3) The applicable image resolution starts at about 400 × 300 pixels, but also smaller images like 160 × 120 pixels enable the recognition of the Bravais lattice type with errors still <5%. Larger images are of course better, but not as much better as their size might suggest.

(4) The projection centre position is the key information. The better it is, the higher the correctness of the derived crystal lattice.

(5) With the help of the Funk transformation, far more than 100 (especially wider) bands can be identified in a single high-quality pattern.

(6) The band positions in combination with the position of the projection centre already fix the lattice parameter ratios as well as α, β and γ. However, the actual values still require knowledge of any single bandwidth in order to determine the lattice and thus the basis vectors from all subsequently selected bandwidths.

(7) Fortunately, the first derivative of a band profile allows the bandwidth to be determined automatically in a suitable way. Subjective errors are thus also reduced to a single value, similar to the drawing of the band positions using a four-line approach, which is valid for all bands.

(8) The double Bragg angles are not perfectly described by bandwidths derived from the first derivative (|Δ2θ| < 5%) but can be satisfactorily corrected by the backscatter coefficient η, which is roughly derivable from the background intensity of the original raw EBSD pattern.

(9) Owing to the small sector covered by the detector compared with the total signal, there is a risk that a number of band profiles will be more or less influenced by neighbouring bands or dominant zone axes as well as by excess-deficiency effects in an unfavourable way. Therefore, it must be assumed that the determined lattice parameters will always contain deviations from the ideal values.

(10) Patterns with exclusively narrow visible Kikuchi bands are a challenge because it is difficult to determine the Bravais lattice type and thus the lattice parameters from a single KD pattern. The band edge profile practically has the width of the Bragg angle, which makes its correct derivation considerably more difficult.

(11) As with all other diffraction techniques, pseudosymmetry can become a serious problem. Superstructure reflections of ordered phases are more likely to be detected in exceptional cases, so that in such cases SAED in a transmission electron microscope is recommended for clarification. In such cases a superlattice will be derived.

(12) Also problematic are phases in which non-equivalent positions are occupied by different elements with almost the same scattering power. But also clearly different scattering powers can lead to wrong Bravais lattice types, if the significantly stronger scattering element occupies a special Wyckoff position and thus suggests a higher symmetry of the lattice.

(13) The detectability of minor lattice distortions is limited by the uncertainty in the position of the projection centre and very small errors in the trace positions.

(14) Local charging needs to be prevented. It not only results in an unpredictable overestimation of the lattice parameters but, together with local magnetic fields, can also lead to image distortions.

Interested persons can obtain the software from the authors on request.

## Supplementary Material

Zipped program with manual. DOI: 10.1107/S1600576721004210/nb5295sup1.zip

## Figures and Tables

**Table 1:** Bravais lattices derived from the KD pattern of cassiterite (SnO_2_, *P*4_2_/*mnm*, η = 0.366, *Z*_at_ = 40.3) shown in Fig. 5 ▸ The first line (bold) refers average values and standard deviations derived from entries of the American Mineralogist Crystal Structure Database (Downs & Hall-Wallace, 2003 ▸). The four lattice descriptions below are possible solutions derived with *CALM*. The resulting angles α, β and γ are listed in italics below the lattice parameters. The numbers in brackets refer to the estimated experimental uncertainty

Lattice	*a*	*b*	*c*	*a*/*b*	*c*/*a*
***tP***	**4.7374 (4)**		**3.1861 (7)**	**1**	**0.6725**

*tI*	4.775 (60)	4.769 (60)	3.217 (40)	0.999	0.674
	*90.2 (1)*	*90.0 (1)*	*89.9 (1)*		
*oF*	3.217 (40)	6.740 (84)	6.757 (85)		
	*89.9 (1)*	*90.2 (1)*	*89.8 (1)*		
*mA*	3.738 (72)	3.217 (40)	6.740 (60)		
	*89.8 (1)*	*90.2 (1)*	*64.7.1 (1)*		
*aP*	3.217 (40)	3.729 (47)	3.738 (47)		
	*100.5 (1)*	*64.7 (1)*	*115.4 (1)*		

**Table 2:** Twenty-three selected cubic phases, the image resolution of the analysed KD patterns and the lattice parameters used for the deviation from the experimentally determined Bragg angle displayed in Fig. 8 ▸

			Resolution (pixels)					
Phase	*Z*_at_	η	320 × 230	400 × 300	673 × 512	800 × 600	1600 × 1200	*a*_o_ (Å)
CaF_2_	12.7	0.172		1				5.463
Al	13.0	0.177			5			4.049
Si	14.0	0.190			2			5.431
CrN	15.5	0.231			1			4.160
FeS_2_	19.3	0.233				10		5.428
Fe_3_O_4_	15.7	0.236		1	3			8.393
Ti (β)	22.0	0.245		2				3.270
Cr_3_Si	21.5	0.250		2				4.564
Cu_3_VS_4_	21.8	0.260				2		5.391
ZnS	23.0	0.275				2		5.408
GaP	23.0	0.280			1			5.451
Ni	28.0	0.295				24		3.524
GaAs	32.0	0.324			1			5.653
NbC	23.5	0.354		3				4.470
InP	32.0	0.379			1			5.869
GaSb	41.0	0.380		1				6.096
Mo	42.0	0.380					3	3.147
PbSe	58.0	0.465		1		1		6.121
Ta	73.0	0.474	3	2				4.420
PbS	49.0	0.475				18		5.914
W	74.0	0.476			1	4		3.158
Au	79.0	0.487			1	6		4.078
Pb	82.0	0.494			3			4.951
