# Supplementary material for: Crystallographic analysis of the lattice metric (CALM) from single electron backscatter diffraction or transmission Kikuchi diffraction patterns
Source: J Appl Crystallogr. 2021 May 28;54(Pt 3):1012–22. doi: 10.1107/S1600576721004210 (PMC8202031; doi:10.1107/S1600576721004210)
Supplement: Supplementary file 1 [file j-54-01012-sup1.zip › CALM-1.4/CALM Software Manual.pdf]

1 Quick guide

1.1 Mouse and keyboard functions

The purely manual definition of band positions and widths by their lattice plane traces requires drawing operations which, due to their complexity, require not only mouse actions but also combinations with the ⇧ and Ctrl keys of the keyboard.

1.1.1 Selection of objects

For each task, an object must first be selected. This is generally done either by pressing

⇧ + 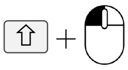 (for **lines**)

or by using the combination of

Ctrl + 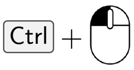 (for **points**).

The activated graphical object (line or point) is then colored in light red. At the same time, the previously selected object changes from light to dark red.

> **Please note:** In the direct projections of the Kukichi pattern, a line (straight line or circular arc) represents a lattice plane trace, while a point indicates the intersection of a lattice plane direction with the imaging screen.
> In the Funk transformation, however, everything is inverse. A point represents (the normal direction of) a lattice plane, while a line (great circle) represents a zone axis on which all lattice plane normals belonging to this zone can be found.

The selection of lines and points works identically in all three projections, whether direct projection of a wide-angle Kikuchi pattern or its Funk transformation.

1.1.2 Declaration of objects

For the declaration or confirmation of new object (lattice directions and lattice planes) a double-click on the left mouse button

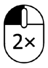

(**confirmation** of a newly defined object)

is used. To define a new intersection point, for example, first two lines have to be selected and secondly the currently shown but only temporary intersection point has to be confirmed by double-clicking with the left mouse button. Objects that have not been declared are automatically rejected.

1.1.3 Calling functions with the keyboard

Moreover, some keys of the keyboard are used to call some functions like Esc to delete the last defined object. All mouse and keyboard interactions are shortly listed in Table 1.

Table 1
Manipulations available in the software. "+" means simultaneously performed actions whereas "&" indicates one or more subsequent actions. Please note that for trace rotation, trace shift and zoom of the pattern the mouse pointer needs to be moved on the displayed pattern (left).

Action	Function
**Mouse functions**	
[⇧] + 🖱 (**ST**)	Selects a line, i.e. line in focus
ST & 🖱	Changes the trace slope (only for the 4 initial traces!)
ST & [⇧] + 🖱	Shifts the trace (only for the 4 initial traces!)
ST & ST	Displays the angle between both (*in Stereographic projection*)
ST & ST & 🖱 2×	Declares a new zone axis
ST & [Ctrl] + 🖱	Defines the width for the selected band, if the mouse pointer is above the *Gnomonic projection*
[⇧] + [Ctrl] + 🖱	Zoom (In & Out) of the Kikuchi pattern if the mouse pointer is above the *Gnomonic projection*
[Ctrl] + 🖱 (**SP**)	Selects an intersection point
SP & SP	Displays the angle between both (*in Stereographic projection*)
SP & SP & 🖱 2×	Defines a new lattice plane by connecting two previously selected zone axes
🖱	performed with the mouse pointer above the window of the reciprocal lattice it rotates the reciprocal lattice and all stereographic projections around the projection normal
**Keyboard functions**	
[Esc]	Deletes the last declared object (trace or point). If a bandwidth is defined, first the bandwidth need to be deleted by [d]
[s]	select the displayed Bragg angle in profile as reference width
[p]	plot the next derived (averaged) Bragg angle (green lines) for the first order (!) and confirm (or omit, cf. [o])
[o]	omit the shown Bragg angle solution gotten after pressing [p]; if you press [o] several times, each time a previously confirmed bandwidths will be deleted
[a]	add band edges (bandwidth) for the white colored trace
[d]	delete the last defined band edges (bandwidth)
[r]	remove the bandwidth of the selected trace as well as all bandwidths derived from it
[1], [2], [3] ... [8]	draw reflection order and reciprocal lattice point for the selected trace
[c]	center the selected $[u\,v\,w]$ or $(h\,k\,l)$ in all rotatable presentations

1.2 Work flow

Please note: The software does not need any installation. Make sure that `CALM v.*.exe` and `glew32.dll` are in the same directory. Possibly missing files like `Gui_default.ini` and `Setup.ini` will be created automatically in this directory.
CALM can be also started from an USB stick or a network drive as long as also `glew32.dll` is there. The Kikuchi patterns can be loaded from any other directory.

1. **Run** `CALM v.*.exe`.
   In case the previously patterns cannot be found or has not yet defined, you possibly need to restart it three times. Finally CALM opens with only the menus visible.
   If you are using a virus scanner, this commonly gives a warning. You need to define a permission for running CALM.

2. **Load Kikuchi pattern**

   - Press the software button [Load] to import a wide-angle Kikuchi diffraction (KD) pattern as bitmap (png, jpg, jpeg, tif, bmp, gif).
   - The **name** of the bitmap file processed **and** its **pixel size** in parenthesis are **shown in the top line** of the user interface.
   - The size of the displayed bitmap has no influence on the size of the *Gnomonic projection* screen, i.e. it is scaled up or down internally.

3. **Input** the **projection center** (PC) position (e.g. derived from a reference pattern)

   Please note: PC is not mandatory for band centre (trace) drawing but necessary for a correct bandwidth definition and Bravais lattice type and lattice parameter determination.
   In other words: If PC is not correct a bandwidth definition is meaningless and results in misleading deviations of the bandwidths.

   - The distances have to be given from the left and the upper edge of the KD pattern, e.g. PC = $(0.500, 0.250, 0.800)$. It means that PC_x und PC_y are running from $0 \ldots 1$ to describe the screen size, independent on the form factor of the screen. PC_z refers to the length of PC_y. The definition is identical to Bruker's pattern simulation software Dynamics.
     For patterns from an Oxford or EDAX system you will need to do a short calculation, i.e.:

     $$PC = (\quad PC_x \quad , \quad 1 - PC_y \quad , \quad PC_Z \cdot F \quad) \qquad \text{(Flamenco)}$$

     $$PC = (\quad PC_x \quad , \quad (1 - PC_y)/F \quad , \quad PC_Z \cdot F \quad) \qquad \text{(Aztec)}$$

     $$PC = (\quad PC_x \quad , \quad 1 - PC_y \quad , \quad PC_Z \quad) \qquad \text{(OIM)}$$

     with the form factor $F = 1344/1024$ (Nordlys), or $F = 1244/1024$ (Symmetry).

     Please note: The confusion for Flamenco or Aztec becomes even bigger as the PC data depends not only on the software used but also on the source

> from which it was taken. Sometimes the numbers are already normalised (cpr files), sometimes they are not (user interface). The here shown equations should work for the non-normalized numbers displayed in the software. Internally (in the `gpd`-file) other coordinates are used which are related to the horizontal screen size assumed as 1.

4. **Define** the used acceleration voltage **HV** of the electrons in kV (in the left bottom screen with the title "Lattice solution"). Otherwise, the derived basis vector lengths a, b and c are wrong since they scale with $1/\sqrt{\mathrm{HV}}$. Bravais lattice type and α, β, γ are, however, correct.

5. **Define FOUR** (initial) **traces**

   - for trace drawing press left mouse key and drag pointer from A (start) to B (end).
   - Define the first four traces in a way that **only two of them share a zone axis** (intersection point). Check this also outside the pattern using the *Stereographic projection* screen.
   - **Chose** for the first four traces **preferably bands** which
     - are far away from each other but well visible,
     - have angles between the traces $> 20°$,
     - form intersection points that describe again visible bands,
     - have a profile as central as possible, (θ asym $\simeq 0$), cf. the *Band profile* screen
     - are narrow (very likely from low-indexed $(h\,k\,l)$).
   - Use the *Funk transform* screen since there all bands are displayed in a way which makes the definition more easier to discover and more reliable.
   - In Funk transform the initial poles can be even grabbed and shifted if the mouse is moved pressing simultaneously: Ctrl + ⇧ + 🖱.

> Please note: You can magnify the KD pattern in the gnomonic projection by ⇧ + Ctrl + 🖱 (mouse wheel). However, you need to be with the mouse pointer in the *Gnomonic projection* screen.

6. **Optimize** (permanently) the **positions of the initial traces** if deviations to band positions become obvious.

   - The aim is to optimize the position of the initial four traces so that all other derived traces also perfectly fit.
   - If not yet active select the checkbox "☑ Band profile" in order to display the band profiles in the *Band profile* screen.
   - **Select** one of the light-green colored **initial traces in gnomonic and stereographic projection** (lines) of the KD pattern by

     ⇧ + 🖱

or **in** the **Funk transformation** (pole) by

Ctrl + 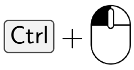.

After selection, active objects change their color into light red.
If the *Band profile* screen is open, additionally the respective band profile appears. If not, move the mouse wheel slightly if the mouse pointer is above the *Gnomonic projection* or the *Funk transform* screen.

- **Tilt** the initial trace around its centre (in the pattern) by using the mouse wheel:

  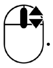.

- **Shift** the initial trace perpendicular to its length by

  ⇧ + 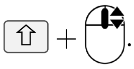.

> **<u>Please note:</u>** Shift and Tilt only work when the mouse pointer is above the "Gnomonic projection‘" or the "Funk transform" screen.

- Very often the position of a trace is perfect when the extrema (green lines) in the *Band profile* screen are symmetric: θ asym $\simeq 0$, e.g. ± 0.012. However, first priority has always the match to all bands. This means, in case of strong excess-deficiency effects bigger θ asym may occur. The size of θ asym also depends on the noise of the signal although CALM already applies a smoothing in steps between 1 and 10. Moreover, there are also several bands which are very asymmetric or falsified by adjacent bands so that a simple search of extrema left and right from $\theta = 0$ does not deliver the according symmetric peaks. This commonly happens when bands show an extreme overlap. For some cases one of the extrema totally disappears, so that such bandwidths should be confirmed only when the Bravais lattice is practically discovered already and remaining bands only should be checked for their match to the found lattice. Therefore, we recommend to declare such bands in a second step.

7. **Derive as many as possible traces** from well-visible bands (typically 60–100) **by connecting two declared zone axes**.

   - Select the radio button " ◉ Declared" below "Poles".

> **<u>Please note:</u>** CALM distinguished between *declared* and *plain* zone axes. **Declared** zone axes mark points which are explicitly defined for the definition of lattice plane traces. According to crystallographic rules these are very often low-indexed zone axis since the combination (vector product) of them results in a low-indexed lattice plane which preferable produce high intensities.
> In contrast, **plain** zone axes are all existing intersections of defined lattice planes.

- In **gnomonic and stereographic projection** of the KD pattern

- **declare new intersection points**, if necessary, by selection of two of the drawn lines using

  ⇧ + 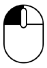

  and double mouse click on one of them

  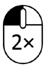

  in order to confirm the declaration.

  > Recommendation: Do not declare too many zone axes, especially not in the KD pattern since for complicated structures very soon they start to disturb. In general, the less zone axes the better. It prevents the definition of higher indexed $[u\,v\,w]$ which increases the probability of misleading trace definitions.

- **Select two** declared **zone axes** by

  Ctrl + 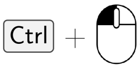.

  A yellow dashed line indicates the just defined trace.

  > Recommendation: You can and should use zone axes outside the pattern!

- **Declare the** temporarily shown **trace** by a double-clicking the left mouse button

  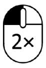

  on one of the intersection points used. The line changes from dashed yellow to solid light red. The respective pole is shown in the *Funk transform* as well as in the *Band profile* screen if they are activated.

  The displayed profiles enables to decide whether the definition is reasonable. It should reflect two clearly visible extrema of the red curve of nearly symmetric positions, i.e. $\theta < 0.15$. This value depends, however, also on the four initial trace positions defined.

  If there is not sufficient match, press Esc to delete the just defined lattice plane. Multiple Esc even may be used to delete the previously declared zone axes.

• In **Funk transformation**

- Prefer the use of the Funk transformation but remember: Poles represent the normals of lattice planes whereas lines (great circles) display zone axes.

– If necessary, **declare new zone axes** (great circles) by selection of two of the defined lattice plane poles

Ctrl + ,

respectively. A red dotted line connecting both poles becomes visible and shows the just defined great circle. A double mouse click on one of the poles confirms the shared zone axis:

2×

whereas a new selection or any other activity cancels the entire definition. The last defined lattice plane and/or zone axes can be deleted by Esc. Each Esc deletes exactly one object.

> Recommendation: Do not declare too many zone axes (great circles). In general, the less great circles are drawn the easier the definition of lattice plane normals as intersection of them.

– **Select two** declared **zone axes** (great circles) by

⇧ + ,

respectively.

– **Declare the** intersection point as new lattice plane **pole** by a double-click of the left mouse button on one of the great circles used:

2×.

The pole is drawn in red (active pole) which changes to blue (inactive) if another pole will be selected. The respective lattice plane trace is also displayed as lines in the gnomonic as well as stereographic projection.
In case of "☑ Band profile", the 1st derivative of the band profile (red curve) enables to decide whether it clearly indicates a band by symmetric θ-positions of maxima and minima, or is possibly a fake band which should be deleted by pressing Esc.

> Please note: The definition of lattice plane traces (band positions) should cover all the bands which are clearly visible. Only then the probability is very low that the following bandwidth description results in a wrong Bravais lattice solution and, therefore, in wrong lattice parameters.
> Remaining bands, which are more speculative since the band edge positions are not symmetric (θ asym), should be added later.

8. **Assign the width of a** (preferably wide) **reference band**.

- Select a lattice plane by its trace in presentations of the pattern, preferably in *Gnomonic projection*, using

  ⇧ +

  or by its pole in *Funk transform* selecting a pole pressing

  Ctrl + .

  Since in *Funk transform* the asymmetric positions of all band edges are shown by bars which partially indicate a imperfect lattice trace alignment, it is a suitable tool to find a perfect band.

  > Please note: Bars are only shown if $0 \leq |\theta\,\mathrm{asym}| \leq 0.3°$. If bigger deviations occur they are caused by other reasons than an imperfect trace alignment. If you want evaluate why no bar is drawn, i.e. whether band edges perfectly match or $\theta\,\mathrm{asym} > 0.3$, select the pole and evaluate its band profile. Commonly, there are only very few bands with perfect match.

- Activate "☑ Band edges" and possibly deactivate "☐ Traces (hkl)" and "☐ Poles [uvw]" in case they disturb.
- There are two different ways for the reference bandwidth definition:
  - **Semi-automatic:** A definition based on the currently selected profile by pressing s on your keyboard. It should be a well centred band ($\theta\,\mathrm{asym} \cong 0$) with a well defined profile. This is more important than the absolute width, i.e., also more narrow bands can be used if wide bands are not available.
  - **Manual:** Definition of the bandwidth using the mouse wheel

    Ctrl + .

    for a selected trace. In any case, the mouse pointer needs to be above the *Gnomonic projection* screen.

    > Please note: This is in fact also the implemented function to adapt all already derived bandwidths equally, e.g. in case it turns out that the reference bandwidth does not show a sufficient correlation to the majority of all other bandwidths.

9. **Combine** intersecting **traces to describe the widths of remaining bands**

- Select "All with" in

  Poles
  ○ Declare
  ◉ All with

  in order to display all zone axes with equal or more intersecting lattice plane traces than defined in the box below. For all intersections $[u\,v\,w]$ with more than two lattice planes $(h\,k\,l)$ change the number to 3:

  $$\geq 3\,(h\,k\,l)$$

This is the standard setting for manual bandwidth definition, i.e. for this task you should not change this value to higher numbers.

- Activate "☑ Band profiles" to display the band profiles.
- Activate the checkbox in "Lattice solution" which is the right field in the lower menu bar. Although this screen might be opened already, you need to activate it in order to get information about possible lattice solutions. This checkbox only enables the real time analysis of the solutions. For less powerful computers leave it deselected.
- **Semi-automatic:** Use `p` (plot) and `o` (omit) to confirm or reject the drawn edges of the selected band on the basis of the displayed band profile.
  - Move the mouse pointer to the *Gnomonic projection* screen.
  - Press `p` to plot the band edge proposal for the next band.
  - When the (first) extrema of the red curve in the *Band profile* screen shows a satisfying **agreement** with the assumed first interference order described by dashed vertical line, you can press `p` to confirm these band edges and plot the proposed edges and profile for the next band.
  - If there is **no agreement** between extrema and dashed vertical lines, `o` need to be pressed which rejects the solution.
    By pressing `p` again the next proposal for a bandwidth description is displayed. This is often for another band.

> **Please note:** This procedure cannot be recalled to continue. Once interrupted, all remaining bands need to be defined manually, or the entire procedure can be restarted by pressing the software button `Reset all`.

> **Please note:** If after pressing `p` the first derived bandwidth is inconsistent with the vertical dashed lines in the band profile screen, a rejection with `o` does not help since the next is the first band again. In this case, after deleting with the software button `Reset all` you need to select another reference bandwidth using `s` and restart the bandwidth assignment.

- **Manual mode:**
  - Activate "Zone" in

    Band edges
    ○ All
    ◉ Zone

    Using "◉ Zone" only traces (including band edges) of this zone are displayed.
  - **Select** one zone axis of preferably three intersecting traces where at least for one band the **bandwidth** is known. This is easy to recognize since CALM automatically colors one band as yellow where the bandwidth is known, and at least one of the other bandwidths is unknown.

> **<u>Please note:</u>** All bands keep their blue color when either no bandwidth is known or all bandwidths are known.

- You can of course change the automated color selection manually. To do so, **select** at any sequence **the intersecting bands** and press

⇧ + 🖱:		for which the **bandwidth** is known (colored yellow),
Ctrl + 🖱:		for which the **bandwidth** is **requested** (colored white)
🖱:		which defines the **translation direction** (colored blue).

- Press a (add) to define the from the yellow and blue traces resulting band edge for the white-colored lattice plane trace, or
- press d (delete) to reject the last defined band edge.

> <u>Please note:</u> A repeated use of d deletes the previously defined band edges one by one.

- The exchange of white and blue colored trace may result in different band edges. The same happens, if blue and yellow can be exchange, i.e. also for the blue trace a bandwidth is defined.

- In most cases, the semi-automated and manual definition of bands will be combined, i.e. first a semi-automated definition of bandwidth is performed which finally delivers the Bravais lattice type and the lattice parameters, and in a second run, for remaining or newly defined traces the band widths are assigned manually.
- If in the main menu "☑ 2D" and/or "☑ 3D" are selected, the reciprocal lattice is shown an can support to find the correct band combination, especially for zone axes with more than 3 intersecting traces.
  More importantly, the final arrangement of reciprocal lattice points need to be inspected in order to discover single points which probably do not match the lattice description, or which are responsible for a bigger unit cell.
- Such single bandwidth can be deleted and, if necessary, redefined. To this end, the respective trace need to be selected. Using r the bandwidth will be deleted from the list. This might delete also some other bandwidths if they were derived from the actually deleted.

10. **Evaluate** the resulting **Bravais lattice type and** the **lattice parameters**

- If the checkbox "☑ Lattice solution" is activated, a separate window lists all possible Bravais lattice descriptions that are conceivable with the currently defined reciprocal lattice points.
- The basis vector lengths a, b and c depend on the High voltage (in kV) assumed in the textbox "HV" .
- Adaptable deviations for the angles (α, β, γ) in degrees for the lattice parameter ratios $(a : b : c)$ in % control the maximum mismatch between theoretical values like $a = b = c$ and $\alpha = \beta = \gamma$ for rhombohedral and derived values from CALM.
- The derived solutions are recomputed again and again, if a new band is added. For low-symmetric phases this can cause to alternative descriptions so that often

the angles between basis vectors may jump to the complementary angle, e.g. 90.4 becomes 89.6.

- The deviations from fixed axes ratios or angles is given for each solution as δ and ϕ. Since for triclinic such conditions do not exist, such values are missing. For all systems with a ratio of 1 between all or some basis vector lengths (cubic, hexagonal, rhombohedral, tetragonal) δ should become 0. For all systems with fixed angles (except triclinic) there is always a ϕ which should be as well 0. The higher the deviation, the lower probability of a correct solution. Please take into account that for systems with more ratios or angles an average value is shown, i.e. the results for each lattice description will be slightly different.
- A double-click on a solution in the *Lattice solutions* screen colors this red and displays the respective unit cell in the *Reciprocal lattice* screen.
- Additionally, the directions of the basis vectors in the *Stereographic projection* screen are indicated as red, green and cyan circles.
- Selecting one of these directions – or also any other $[u\,v\,w]$ or $(h\,k\,l)$ – will generate a projection along this direction, if [c] (center) is pressed. Then all rotatable projections will be centred according to the selected direction.
- After activation of "☑ Indices" all intersection points are indexed according to the selected Bravais lattice solution. The is valid for points representing $[u\,v\,w]$ but also for point indicating $(h\,k\,l)$ in *Funk transform.*
- The lattice solution can be deactivated either by selecting another description, or by double-click on an empty area. Then all solutions are deactivated and indices disappear.
