# Supplementary figures and images for: Crystallographic analysis of the lattice metric (CALM) from single electron backscatter diffraction or transmission Kikuchi diffraction patterns
Source: J Appl Crystallogr. 2021 May 28;54(Pt 3):1012–22. doi: 10.1107/S1600576721004210 (PMC8202031; doi:10.1107/S1600576721004210)

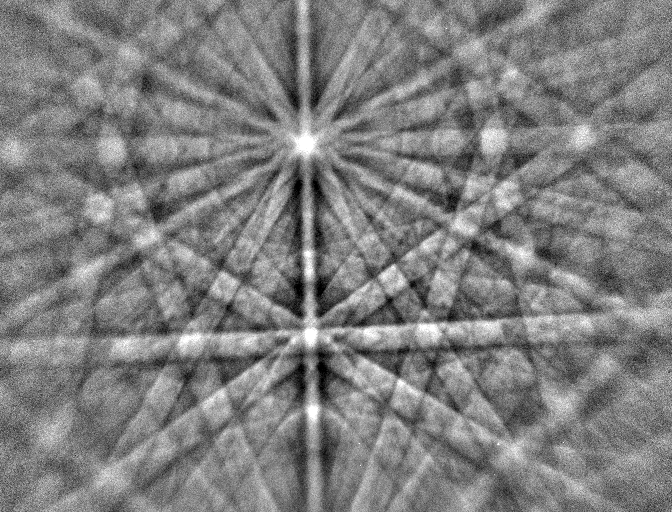

Supplement: Supplementary file 1 [file j-54-01012-sup1.zip › CALM-1.4/PATTERNS/Ag(x)Sn.png]

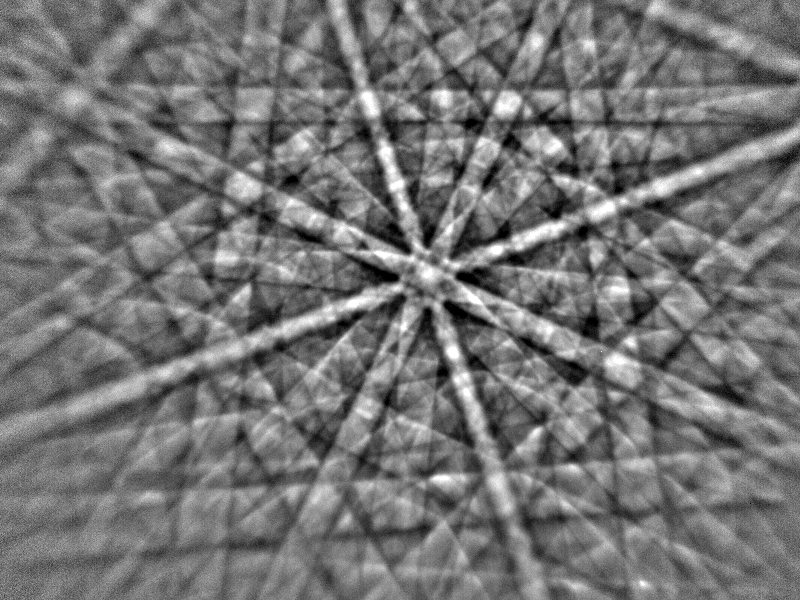

Supplement: Supplementary file 1 [file j-54-01012-sup1.zip › CALM-1.4/PATTERNS/Fe_01_bcc.png]

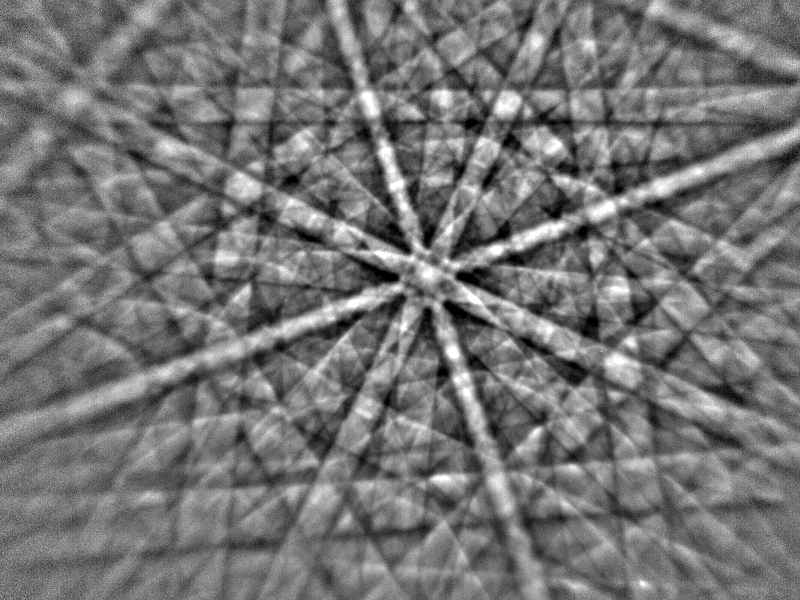

Supplement: Supplementary file 1 [file j-54-01012-sup1.zip › CALM-1.4/PATTERNS/Fe_01_bccWithTraces.png]

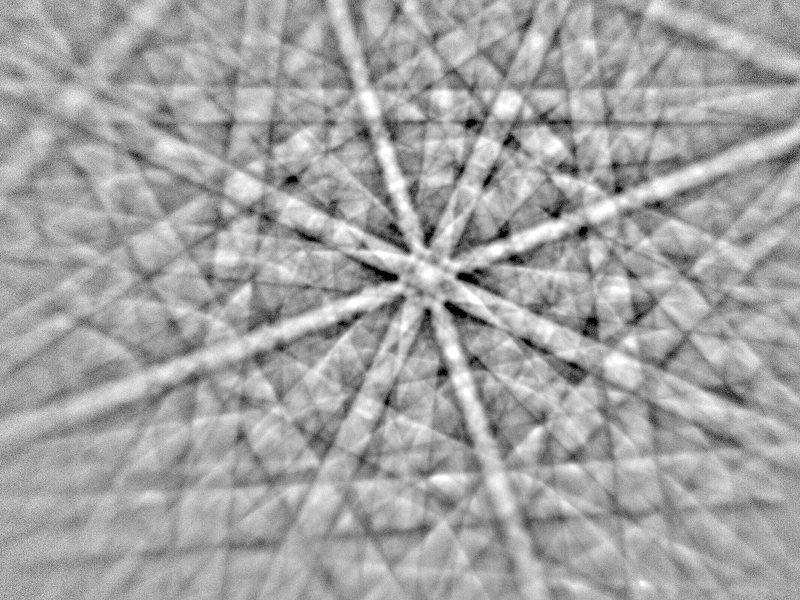

Supplement: Supplementary file 1 [file j-54-01012-sup1.zip › CALM-1.4/PATTERNS/Fe_01_bcc_with bands.png]

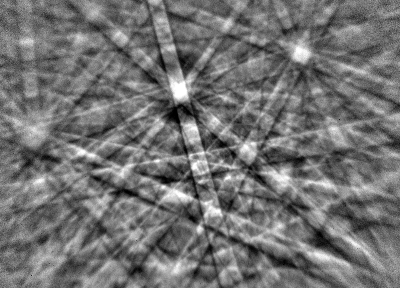

Supplement: Supplementary file 1 [file j-54-01012-sup1.zip › CALM-1.4/PATTERNS/Korund_01.png]

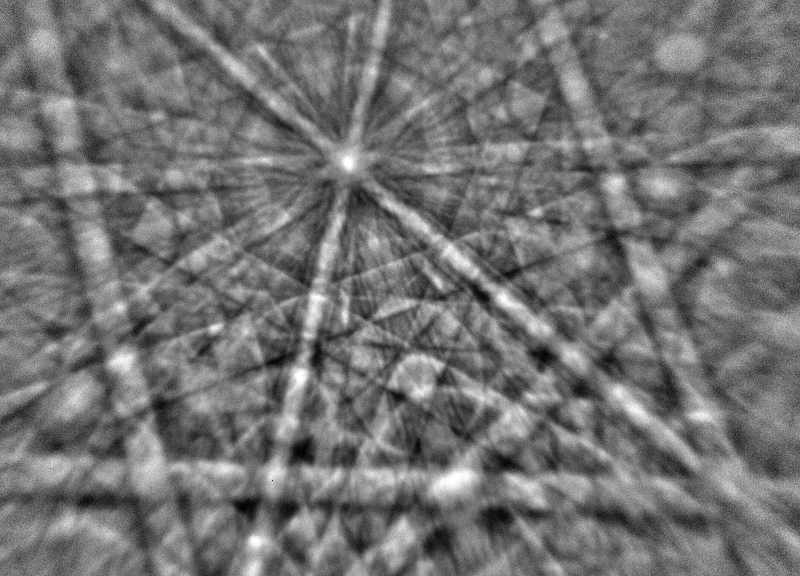

Supplement: Supplementary file 1 [file j-54-01012-sup1.zip › CALM-1.4/PATTERNS/Rammelsbergite_01.png]
